# Cholesterol versus Inflammation as Cause of Chronic Diseases

**DOI:** 10.3390/nu11102332

**Published:** 2019-10-01

**Authors:** Sunil K. Panchal, Lindsay Brown

**Affiliations:** 1 Functional Foods Research Group, University of Southern Queensland, Toowoomba, QLD 4350, Australia; Sunil.Panchal@usq.edu.au; 2 School of Health and Wellbeing, University of Southern Queensland, Toowoomba, QLD 4350, Australia

**Keywords:** Chronic disease, cholesterol, inflammation, diet, statin, cardiovascular

Tsoupras and colleagues have postulated, in a recent review in *Nutrients*, that the key to reducing the incidence of cardiovascular disease is to control the activities of inflammatory mediators such as platelet-activating factor (PAF) by diet, exercise, and healthy lifestyle choices [1]. We agree with this sensible conclusion, which is based on the extensive scientific literature. The authors argue that their proposal is contrary to the lipid (or cholesterol) hypothesis. This supposes that the reduction of plasma lipid concentrations will reduce new incidents of cardiovascular disease, a concept developed over many decades from the recommendation in 1976 that a combined drug/diet regimen in a study with large numbers of male hyperlipidemic subjects would be necessary to test the accuracy of the hypothesis [2]. Following extensive studies with the statins as lipid-lowering drugs, the accumulation of evidence for the lipid hypothesis was then presented in 2006 with Steinberg concluding that the controversy was solved by the results of these studies, stating that “The cholesterol controversy could have been resolved much earlier if all of us had looked at all of the evidence” [3]. However, this conclusion on the lipid hypothesis continues to arouse passionate arguments, as shown in the review by Tsoupras and colleagues [1] and in other recent reviews [4].

Chronic disease of the cardiovascular system is not just a modern phenomenon. Atherosclerosis has been identified using computed tomography in ancient Egyptian, Peruvian, and Native American mummies with the Egyptian Princess Ahmose-Meryet-Amon who lived around 1580–1550 B.C. as the earliest human known to have had coronary artery disease [5,6]. These findings led to the question “Might the chronic inflammatory load of ancient times secondary to infection have resulted in atherosclerosis?” [5]. The scientific basis of inflammation in atherosclerosis also has a long history. The influential European pathologists of the 19th century, Rudolf Virchow and Carl von Rokitansky, described inflammatory changes in atherosclerosis, with Virchow supporting the primary role of inflammation while von Rokitansky argued that incorporated mural thrombosis was the key pathogenic process in atherosclerosis [7]; both mechanisms are incorporated in current concepts of the development of atherosclerosis [8]. Insights provided by molecular and cellular biology into the complexity of atherosclerosis have given a better idea of the mechanisms that link risk factors such as low-density lipoproteins (LDL), hypertension, cigarette smoking, diabetes, and inflammation to the clinical disease [9].

What is the role of cholesterol in cardiovascular health and disease? As noted by Tsoupras and co-authors [1], cholesterol is essential for the normal function of cells including the structural makeup and fluidity of cell membranes, signal transduction, intracellular transport, nerve conduction, signaling pathways through lipid rafts and caveolae, and, as the precursor for vitamin D, steroid hormones, and bile salts. Most of the cholesterol in humans comes from endogenous production, supporting the finding by Ancel Keys and co-workers that changing dietary cholesterol in humans produced minor effects on serum cholesterol concentrations [10]. As lipid-soluble compounds, cholesterol and triglycerides are transported in lipoproteins that are necessary for absorption of dietary lipids from the small intestine, transport of lipids to peripheral tissues, and transport of lipids back to the liver and intestine. LDL that transport most of the cholesterol are considered pro-atherogenic. The connection between cholesterol and atherogenesis was first reported in 1913 by Anitschkow and Chalatow in rabbits fed egg yolk [11]. But do dietary cholesterol intakes and serum cholesterol concentrations relate to human atherosclerosis? In 1958, Ancel Keys and his team started the Seven Countries Study (SCS) which reported marked differences in rates of coronary heart disease events and deaths among cohorts and strong associations of average dietary features and blood cholesterol concentrations with those rates [12,13]. The controversies from these conclusions are on-going [14]. Further, the role of dietary fat intakes on cardiovascular disease continues to be argued. As a recent example, the Prospective Urban Rural Epidemiology (PURE) study results from 135,335 subjects over five continents that intake of total fat and individual types of fat was unrelated to cardiovascular disease or mortality [15] have been labeled as “misleading” [16].

If cholesterol is important in cardiovascular disease, then selective inhibition by the statins of HMG-CoA reductase, a rate-limiting step in endogenous cholesterol synthesis, should decrease cardiovascular risk. This result has been found in many, but not all, studies. It is now 25 years since the publication of the Scandinavian Simvastatin Survival Study (4S) showing that simvastatin reduced LDL-cholesterol by 35% with a relative risk of death of 0.70 with 5-year treatment in 4,444 subjects with angina pectoris or previous myocardial infarction and serum cholesterol concentrations of 5.5 to 8 mmol/L [17]. Statin therapy is recommended for primary prevention of stroke with each decrease of 1 mmol/L in serum cholesterol concentrations equating to a reduction in relative risk by 21% [18]. Statin therapy reduced the risk of heart failure in patients with cardiovascular risk factors but without heart failure; however, statins did not reduce rates of cardiovascular death in patients with heart failure [19]. In contrast, Cochrane meta-analyses of cholesterol lowering with statins in peripheral arterial disease of the lower extremities in acute coronary syndromes reported no benefit [20,21]. Similarly, the lack of an association between LDL-cholesterol and mortality in the elderly has been reported through systematic review [22]. Importantly, statins produce a range of pleiotropic effects in addition to inhibition of cholesterol synthesis, especially to reduce inflammatory markers such as C-reactive protein, which may be important in reducing the formation of atherosclerotic plaque [23]. However, statins do not completely prevent atherosclerotic vascular disease. If atherosclerosis is a complex inflammatory disorder, then anti-inflammatory therapy on top of intense lipid-lowering treatment should lower the risk of acute cardiovascular events. This has now been proved with the Canakinumab Anti-inflammatory Thrombosis Outcomes Study (CANTOS) trial with targeted inhibition of interleukin (IL)-1β with canakinumab [24]. Further, the residual risk after canakinumab may be related to the persistence of high concentrations of other inflammatory cytokines such as IL-6 and IL-18 [25]. Novel anti-inflammatory compounds are in Phase II and III trials for treatment of atherosclerosis [26]. The modulatory role of inflammation is now unambiguous, showing that “inflammation provides a pathway that mechanistically links alterations in traditional risk factors and modifications in the biology of the artery wall that give rise to atherosclerosis and its complications” [27].

If inflammation is critical in atherosclerosis, then resolving chronic inflammation could be the next approach to decrease atherosclerotic risk. Under normal physiological circumstances, inflammation resolves after the clearance of infection or injurious agents; however, in chronic conditions, the inflammatory response continues, leading to continuing tissue damage [28]. Resolution of inflammation is controlled by specialized proresolving mediators such as lipoxins, resolvins, protectins, and maresins [29]. The development of selective compounds that are effective in promoting resolution of inflammation will further support the role of inflammation in atherosclerosis.

The formation of inflammatory atherosclerotic lesions follows the accumulation of lipids in the vascular wall from the circulation. Plasma cholesterol concentrations may then be a biomarker rather than the cause for the development of atherosclerosis, as treatment of inflammation, either prevention or reversal, is the key therapeutic aim. Chronic inflammation has been proposed as a major player in the development and progression of other chronic diseases [28,30,31,32]. Further, targeting chronic inflammation has been suggested as an intervention to reduce aging-related complications and improving tissue repair [28,33].

Chronic inflammation may be managed by lifestyle interventions and dietary modifications to restrict progression of tissue damage. Many dietary interventions have been suggested to improve outcomes in chronic diseases [34,35,36,37,38,39,40,41,42,43]. The main aim of these dietary interventions is to lower inflammation in the body. Low prevalence of cardiovascular mortality in the Mediterranean cohorts of SCS was correlated to their lifestyle and Mediterranean diet [44,45]. The Prevención con Dieta Mediterránea (PREDIMED) trial provided strong evidence for vegetable-based Mediterranean diets with increased unsaturated fat and polyphenols in the prevention of cardiovascular disease [46]. The PREDIMED trial used the Dietary Inflammatory Index to show an increased risk of cardiovascular events with a proinflammatory diet [47]. The Okinawan diet, characterized by low caloric intake; high consumption of vegetables (particularly root and green–yellow vegetables), legumes, and fish products; low consumption of meat products and dairy products; low fat intake; low-glycemic index carbohydrates; high fiber intake; and moderate alcohol consumption, modulates insulin signaling and inflammatory pathways [43]. The Okinawan diet has also been suggested to reduce risks for aging-associated chronic diseases and to promote longevity [43]. The Nordic diet, which is similar in composition to the Mediterranean diet but less researched for health benefits, has shown anti-inflammatory effects [42].

These diets and others such as DASH (Dietary Approaches to Stop Hypertension) and portfolio diets have the same aim of reducing systemic inflammation [43]. The reduction in chronic inflammation would reduce the tissue damage to prevent disease progression [33]. Improvement in tissue status, along with anti-inflammatory effects, would improve overall metabolic health of an individual. As an example, the Mediterranean diet decreased hepatic fat content, a risk factor for metabolic syndrome, type 2 diabetes, and coronary heart disease [48]. Complete removal of dietary meat as in vegetarian diets reduces body mass, improves plasma lipid profile, gut microbiota, and insulin sensitivity, and decreases the incidence of cardiovascular disease, stroke, metabolic syndrome, arteriosclerosis, diabetes, and cancer. However, the vegetarian diet has been associated with development of hyperhomocysteinemia, protein deficiency, anemia, decreased creatinine content in muscles, menstrual disruption in women, and increased risk of osteoporosis and bone fractures [49,50,51,52,53,54,55,56,57,58]. The role of exercise and physical activity in obesity reduction and management have been well established [59,60]. With an increased caloric intake, increasing energy expenditure through exercise and physical activity will help maintain the weight status [60]. Moreover, a meta-analysis suggested that engaging in exercise training decreased C-reactive protein regardless of the age or sex of the individual [61]. This meta-analysis also concluded that the improvements in C-reactive protein were greater with a decrease in body mass index or % body fat [61]. Other studies have also reported anti-inflammatory benefits of physical activity and exercise [62,63,64,65,66], which would certainly help reduce the risk of chronic diseases.

Tsoupras and colleagues have thoroughly reviewed the role of PAF in controlling inflammation. They have clearly listed the roles PAF can play in normal physiological conditions and in atherosclerosis and cardiovascular diseases [1]. The question now is whether PAF is the most important inflammatory mediator that can be controlled for preventing chronic inflammatory diseases. The answer to this question is likely ‘no’ as there are many other regulators of inflammatory pathways [66,67,68,69,70,71]. Thus, identifying and targeting selective inflammatory pathways related to the cause and stage of disease development in groups of individuals could provide a more effective solution for treating inflammation-induced chronic diseases.

In conclusion, we agree that the logical way to decrease the inflammation-induced risk of chronic diseases such as atherosclerosis is to decrease pro-inflammatory inputs. Using drugs, diets, and exercise could provide the anti-inflammatory mechanisms to achieve these targets. However, consideration of the wide range of inflammatory mediators in addition to PAF is needed to reduce the risk in populations with multifactorial causes for the initiation of inflammation. Finally, while the concept of functional foods allows the testing and eventual use of foods for chronic disease prevention and management, attributing the saying to Hippocrates that “Let food be thy medicine and medicine be thy food” appears to be a historical, if oft-repeated, misquotation [72].
